# Dissecting Heterogeneity Reveals a Unique BAMBI^high^MFGE8^high^ Subpopulation of Human UC‐MSCs

**DOI:** 10.1002/advs.202202510

**Published:** 2022-11-14

**Authors:** Hongwei Chen, Xin Wen, Shanshan Liu, Tian Sun, Hua Song, Fang Wang, Jiayue Xu, Yueyang Zhang, Yuanjin Zhao, Jia Yu, Lingyun Sun

**Affiliations:** ^1^ Department of Rheumatology and Immunology The Affiliated Drum Tower Hospital of Nanjing University Medical School Nanjing 210008 P. R. China; ^2^ Department of Biochemistry Institute of Basic Medical Sciences Chinese Academy of Medical Sciences (CAMS) and School of Basic Medicine Peking Union Medical College (PUMC) Beijing 100005 P. R. China; ^3^ School of Basic Medicine and Clinical Pharmacy China Pharmaceutical University Nanjing 211198 P. R. China

**Keywords:** BAMBI^high^MFGE8^high^ umbilical cord‐derived mesenchymal stem cells, differentiation, heterogeneity, mesenchymal stem cells, systemic lupus erythematosus

## Abstract

Mixed human umbilical cord‐derived mesenchymal stem cells (UC‐MSCs) are widely applied in clinical trials to treat various diseases due to their multipotent differentiation potential and immune regulatory activities. However, the lack of a clear understanding of their heterogeneity hampers their application to precisely treat diseases. Moreover, few studies have experimentally authenticated the functions of so‐called UC‐MSC subpopulations classified from scRNA‐seq samples. Here, this work draws a large‐scale single‐cell transcriptomic atlas and identified three clusters (C1, C2, and C3), representing the primed, intermediate, and stem statuses individually. The C1 and C3 clusters feature higher expression of cytokines and stemness markers, respectively. Surprisingly, further experimental assays reveal that the BAMBI^high^MFGE8^high^ C1 subgroup has a unique phenotype, distinct transcriptomic profile, and limited adipogenic differentiation potential but compromised immunosuppressive activity in vitro and in vivo in lupus mice. Thus, this work is helpful to clarify the nature of human UC‐MSCs and to choose optimal MSC types to treat specific diseases in the future.

## Graphical abstract

Large‐scale scRNA‐seq uncovers a unique BAMBI^high^MFGE8^high^ subpopulation hidden in heterogeneous human UC‐MSCs. It has special features in terms of phenotype, distinct transcriptomic profile, limited adipogenic differentiation potential, and unconventional immunological properties. This study is helpful to clarify the nature of human UC‐MSCs and to facilitate the use of appropriate MSC types to treat specific diseases.

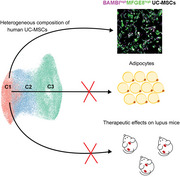

## 1 Introduction

Mesenchymal stem cells (MSCs) are self‐renewing precursor cells capable of differentiating into osteocytes, adipocytes, chondrocytes, and some other cellular types.^[^ ^1^ ^]^ They can be derived from but not limited to bone marrow, umbilical cord, and adipose tissues.^[^ ^2^ ^]^ MSCs have been widely applied in clinical trials to treat various diseases, including autoimmune diseases,^[^ ^3^ ^]^ degenerative disorders,^[^ ^4^ ^]^ cancers,^[^ ^5^ ^]^ and the COVID‐19 pandemic,^[^ ^6^ ^]^ due to their broad differentiation potential to repair damaged tissues and effective immunomodulation activities.^[^ ^2^ ^]^ MSC therapy has been proven to reset the immune tolerance state in autoimmune diseases. For instance, human umbilical cord‐derived MSC (UC‐MSC) transplantation augments peripheral blood tolerogenic CD1c^+^ dendritic cells in refractory systemic lupus erythematosus (SLE) patients,^[^ ^3a^ ^]^ while bone marrow MSCs (BM‐MSCs) can increase CD4^+^CD25^+^FoxP3^+^ Treg cells in systemic sclerosis and experimental colitis.^[^ ^7^ ^]^ Although MSC application shows promising therapeutic effects, especially in preclinical animal models, most registered clinical trials have turned down primary expectations.^[^ ^2^ ^]^ Indeed, 40% of SLE patients with MSC infusion showed no clinical response after 12 months of follow‐up visits from a multicenter clinical study.^[^ ^8^ ^]^ Among the many factors influencing eventual clinical efficacy, the heterogeneous composition of the incompetent MSC population is assumed to be an indispensable or perhaps a prerequisite contributor to the failure of MSC therapy. However, which subtype interferes with the effective outcome of MSC transplantation for SLE is unknown because the nature of MSC heterogeneity is still not very clear.

MSC heterogeneity refers to its cellular variability in terms of molecular fluctuation state, morphology, differentiation capacity, therapeutic function, etc., which comes from not only donor sources but also distinct cell subtypes within a mixed population. The heterogeneity of MSCs is recognized by the existence of MSCs with different morphologies (spindle vs flat or short vs long) and sizes simultaneously in culture. The phenomenon of heterogeneity was proven with single‐cell clones by time‐lapse analysis,^[^ ^9^ ^]^ together with their distinct transcriptional profile and preferential differentiation potentials by bulk‐cell sequencing.^[^ ^10^ ^]^ By using ATAC‐seq, Ho et al. found that chromatin accessibility identifies the diversity of MSCs from different origins.^[^ ^11^ ^]^ Based on single‐cell RNA sequencing (scRNA‐seq) with a small quantity of samples, human UC‐MSCs are considered to have limited heterogeneity with unique gene expression.^[^ ^12^ ^]^ Other work applying the 10× Genomics sequencing platform highlights distinct subpopulations within UC‐MSCs in vitro and in vivo with specified transcriptomic features under bioinformatics analysis.^[^ ^13^ ^]^ These scRNA‐seq results show that conventional MSC cell surface markers (CD44, CD73, CD90, CD105, CD106, CD271, CD146, CXCR4, etc.)^[^ ^14^ ^]^ are not sufficient to isolate different MSC subgroups. Unfortunately, few studies have further authenticated the true functions of these so‐called UC‐MSC subpopulations with subsequent experiments, such as differentiation potential and immune regulatory activities. In addition, a lack of clear knowledge of UC‐MSC heterogeneity still hampers the choice of desired optimal cell types for clinical therapeutic applications. Thus, it is essential to comprehensively understand the landscape of UC‐MSC heterogeneity by using a large quantity of samples and to explore novel strategies to directly or indirectly obtain appropriate cellular subtypes within the population for specific diseases.

In this study, a comprehensive human UC‐MSC single‐cell transcriptomic atlas was established based on the sequencing of 129633 (61974 from P0 and 67659 from P7) cells from six donors by using a 10× Genomics platform. Three subclusters (C1–C3) were identified in the heterogeneous UC‐MSCs, of which the C1 subgroup was successfully pooled with a novel BAMBI^high^MFGE8^high^ FACS sorting strategy. Further characterization of the BAMBI^high^MFGE8^high^ cells revealed that they are a compromised adipogenic and immunosuppressive subpopulation with unique features in heterogeneous UC‐MSCs. These results are helpful to uncover the basic characteristics of MSCs and to apply precise MSC types to improve the clinical efficacy of MSC therapy.

## 2 Results

### 2.1 Single‐Cell Transcriptomic Heterogeneity of UC‐MSCs

**Figure 1:**
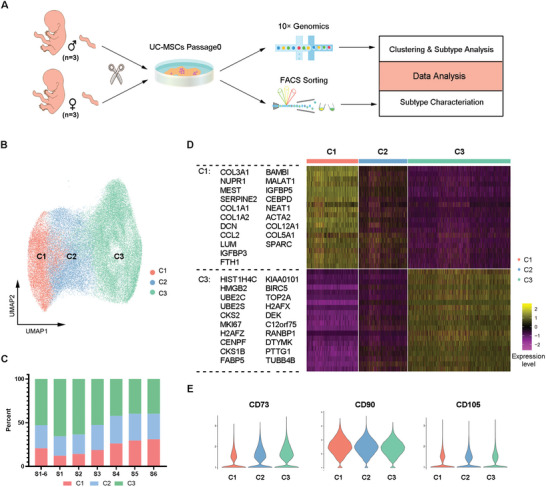
Single‐cell transcriptomic analysis revealed the heterogeneity of UC‐MSCs at P0. A) Workflow of the study. B) UMAP visualization of single cell clusters of UC‐MSCs at P0. C) Distribution of the three subgroups among six donors. D) Heatmap depicting the expression levels of the top 20 differentially expressed genes in the subgroups of UC‐MSCs at P0. E) Violin plots showing CD73, CD90, and CD105 expression in P0 UC‐MSC clusters.

To understand how UC‐MSCs are formed by different subgroups, we analyzed the single‐cell transcriptomic data of primary (P0) human UC‐MSCs derived from Wharton's jellies of umbilical cords from six newborns, including three females and three males (**Figure** **1**A, Tables S1 and S2, Supporting Information). After data processing, a total of 58353 cells and 55514 cells passed quality control from P0 and P7, respectively, without cell cycle regression considering the overlap of cell cycle‐ and stemness maintenance‐related genes. We merged all the single‐cell transcriptomes from P0 to show a primary human UC‐MSC heterogeneity atlas by UMAP. UC‐MSCs were shown to be a heterogeneous population with 10 subgroups by the top 500 highly variable genes (Figure S1A,B, Supporting Information). Subgroup 9, covering less than 1% of total cells, was further filtered out since it was predominantly observed only in donor S5 with enriched genes responding to decreased oxygen levels and the p53 signaling pathway, implying possible cellular injury during the manipulation process for library construction (Figure S1C, Supporting Information). Next, detection of the ratio of the remaining 9 clusters showed consistency of these subgroups across all 6 donors with fraction variations for specific clusters, indicating universal cellular heterogeneity with minor differences between individuals (Figure S1D, Supporting Information).

Upon examining the distribution and heatmap of the top 10 highly expressed genes in the subgroups, many enriched genes were shared by certain subgroups (Figure S1E,F, Supporting Information). We next performed a principal component analysis (PCA) and correlation analysis on the 9 subgroups to discriminate three main clusters (Figure S1G,H, Supporting Information). Thus, such secondary subgroups were merged together to correct the UC‐MSC population to three main clusters named C1, C2, and C3 (Figure 1B). In accordance with previous observations, the composition of the C1–C3 clusters was compatible with minor fluctuations among the six donors (Figure 1C).

To characterize the molecular heterogeneity of UC‐MSCs at P0, we further examined the differentially expressed genes of the three clusters (Data S1, Supporting Information). The top 20 differentially expressed genes (DEGs) in C1 and C3 clearly separated the two clusters (Figure 1D). However, there were no significantly highly expressed genes in C2, which is speculated to be an intermediate population between C1 and C3. To understand whether there is any biased expression of MSC markers in heterogeneous UC‐MSCs, we checked the cell surface markers defined by ISCT^[^ ^14^ ^]^ across C1–C3 clusters. The expression of CD73, CD90, and CD105 was confirmed but without dramatic specificity in any of the three clusters. Only CD73 expression was slightly increased from C1 to C3 (Figure 1E). None of the other previous MSC markers (CD13, CD29, CD44, CD140a, CD164, CD200)^[^ ^13a^ ^]^ showed expression differences to specifically separate the three subclusters (Figure S2A, Supporting Information). Our further examination of recently proposed MSC surface markers^[^ ^11^, ^12^, ^15^ ^]^ to distinguish UC‐MSC populations indicated that CD142 expression was constant within the C1–C3 clusters (Figure S2A, Supporting Information), while others were gradually downregulated (CD140b/PDGFRB) or upregulated (CD166/ALCAM and CD168/HHMR) from C1 to C3. Although CD168/HHMR expression was relatively upregulated in C3, this marker seemed incapable of reliably separating the three clusters because its expression was strongly cell cycle‐dependent.^[^ ^11^ ^]^

Eventually, to ensure that the three classified UC‐MSC clusters were not solely cell cycle determined, we verified the expression pattern of representative genes (BAMBI, CXCL2, IGFBP5, FABP5, BIRC5) in the cells with cell cycle regression, and the results showed that they were indeed still constrained in specific cell clusters, which was the opposite case for cell cycle marker genes (PCNA for S phase and CCNF for G2/M phase, Figure S2B,C, Supporting Information and Data S2, Supporting Information). Therefore, UC‐MSCs at P0 are made of three distinct subpopulations (C1, C2, and C3) at the single‐cell transcriptomic level.

### 2.2 Single‐Cell Transcriptomic Signature of UC‐MSC Subgroups

**Figure 2:**
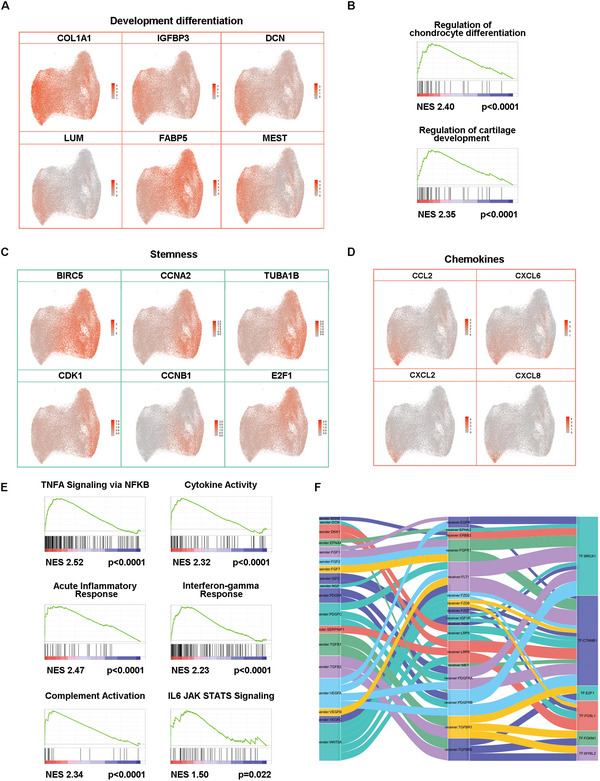
Single‐cell transcriptomic signature of P0 UC‐MSC subgroups. A) Feature plot showing differentiation‐related gene expression levels in P0 UC‐MSCs. B) GSEA showing chondrogenic‐related signaling pathways upregulated in P0 UC‐MSCs from C1 compared to C3. C) Feature plot showing the stemness‐related gene expression level of P0 UC‐MSCs. D) Feature plot showing chemokine‐related gene expression levels in UC‐MSCs at P0.E) GSEA showing inflammatory‐related signaling pathways upregulated in C1 compared to C3 in P0 UC‐MSCs. F) Cell communication between C1 and C3.

To judge whether there was differentiation bias among the three clusters, we predicted their developmental fate according to the mRNA expression levels of osteogenic (COL1A1, IGFBP3), chondrogenic (DCN, LUM), and adipogenic (FABP5, MEST) markers chosen from the top 20 DEGs (**Figure** **2**A). Relatively, the highest expression of chondrogenic and osteogenic genes was revealed in C1. For adipogenesis, there was higher expression of its positive regulator (FABP5) in C3 and negative regulator (MEST) in C1. Further gene set enrichment analysis (GSEA) results also verified the higher chondrogenic differentiation tendency for C1 (Figure 2B). Thus, C1 is presumably more primed for osteochondrogenic differentiation with adipogenic inhibition, while C3 may have an enhanced ability to produce adipocytes. In terms of stemness, the C3 subgroup was intensively enriched with stemness markers (BIRC5, CCNA2, TUBA1B, CDK1, CCNB1, E2F1) compared to the C1 and C2 clusters (Figure 2C), indicating its strengthened self‐renewal stem cell state. In any case above, the C2 cluster stands between C1 and C3, suggesting its intermediate condition.

To illustrate the potential immune regulatory function of UC‐MSC subgroups at P0, we explored highly expressed genes across C1–C3 and found that C1 featured higher expression of cytokines (CCL2, CXCL2, CXCL6, and CXCL8) than C2 or C3 (Figure 2D). Consistent with the gene expression level, GSEA of the C1 DEGs revealed multiple immune‐related pathways, including TNF*α* signaling via NFКb, interferon response, IL6‐JAK‐STATS signaling, cytokine activity, complement activation and acute inflammatory response (Figure 2E), suggesting a susceptible state of immunity of C1 compared to C3 in culture, where C1 is likely to have unconventional anti‐inflammatory effects upon regulating immune cells as known previously.^[^ ^16^ ^]^

To more comprehensively dissect the difference in functional signaling pathways between C1 and C3, we performed pathway enrichment analysis. The results reveal that C1 UC‐MSCs are enriched in responses to TGF*β* and hormones, as well as interleukin (IL‐4, IL‐13, IL‐17, and IL‐18) pathways, many of which drive chemokine expression and are involved in immunomodulation. In addition, C1 was also enriched in ECM organization, blood vessel development and collagen fibril organization, indicating the regulation of developmental differentiation, tissue regeneration and stress response. In contrast, the pathway enrichment analysis in C3 shows that this subset is mostly enriched in the regulation of cell cycle processes, RHO GTPase effectors, triglyceride catabolism and the regulation of stem cell proliferation, indicating their relatively unprimed stem state (Figure S2D, Supporting Information).

Finally, we constructed a protein interaction network enriched in C1 and C3, and notably found that some differentially expressed genes in C1 (CEBPD, JUNB, FOS, and CDKN1A) and C3 (BIRC5, HMGA2, FABP4, and NOTCH1) had interactions with each other, suggesting their potential intercellular interaction (Figure S2E, Supporting Information). In addition, we examined cell communication from C1 to C3 and found that FGF, TGF, PDGF, and VEGF signaling members may influence cellular communication between C1 and C3 (Figure 2F).

### 2.3 BAMBI^high^MFGE8^high^ Cells Enriched in the C1 Subgroup in Both Primary and Expanded UC‐MSCs

**Figure 3:**
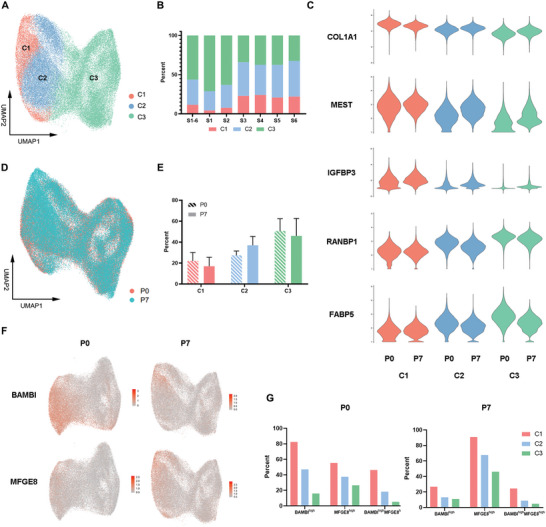
BAMBI^high^MFGE8^high^ UC‐MSCs were enriched in the C1 subgroup of P0 and P7. A) UMAP visualization of single cell clusters of UC‐MSCs at P7. B) Distribution of the three subgroups of UC‐MSCs at P7 among six donors. C) Violin plots showing specific higher expression genes in P7 UC‐MSC clustering. D) UMAP visualization of P0 and P7 UC‐MSCs clustering together. E) Distribution of the three subgroups between P0 and P7 UC‐MSCs. F) Feature plot showing high BAMBI and MFGE8 expression levels in P0 and P7 UC‐MSCs. G) Proportion of BAMBI^high^, MFGE8^high^, and BAMBI^high^ MFGE8^high^ cells in the subgroups of P0 and P7 UC‐MSCs.

By using the same method as that used at P0, we analyzed UC‐MSCs at P7, which are conventionally used to treat SLE patients,^[^ ^3a^ ^]^ to understand the heterogeneity status of long‐term maintained UC‐MSCs. The results showed that UC‐MSCs (P7) could be clustered into 11 subgroups after filtering out subgroup 11 since they covered less than 1% of the total cells and were enriched with muscle/actin‐related pathways (Figure S3A–C, Supporting Information). Similar to UC‐MSCs at P0, UC‐MSCs at P7 also consisted of three main clusters, with the secondary subgroups merged by PCA and correlation analysis (**Figure** **3**A and Figure S3D,E, Supporting Information). The distribution of three clusters for P7 remained variable across six donors (Figure 3B and Figure S3B, Supporting Information). Likewise, we inspected mostly differentially expressed signature genes in P7 (Data S3, Supporting Information) and found expression trends compatible with those in P0 (Figure 3C and Figure S3F, Supporting Information). When pooling all the cells, we only observed a minor variation in the homogeneity between P0 and P7 (Figure 3D and Figure S3G, Supporting Information). Notably, we detected an increase in the C2 fraction but a decrease in C1 and C3 after long‐term culture in vitro (Figure 3E), suggesting that current culture conditions may converge UC‐MSC subgroups.

As none of the CD markers seem specific to separate C1–C3 clusters at will (Figure 1E and Figure S2A, Supporting Information), we explored other transmembrane proteins as potential markers to sort the cell subtypes. To our surprise, both BAMBI and MFGE8 expression levels showed high specificity across UC‐MSCs at P0 and P7 (Figure 3F and Figure S3H, Supporting Information). BAMBI is involved in regulating adipogenesis and Treg/Th17 differentiation.^[^ ^17^ ^]^ MSC‐derived MFGE8 plays an active role in antifibrosis and wound healing.^[^ ^18^ ^]^ Hence, these results implied that BAMBI and MFGE8 may be used as candidate markers. The fraction of BAMBI expression at high mRNA levels gradually decreased at P0, with 82.42%, 47.02%, and 15.94% for C1, C2, and C3, respectively (Figure 3G). MFGE8 shows a similar expression pattern to BAMBI. In the C1 cluster, nearly half of the cells were BAMBI^high^MFGE8^high^ cells, while only 18.31% and 5.26% of these cells were in C2 and C3, respectively. For the UC‐MSCs at P7, BAMBI^high^MFGE8^high^ cells were also enriched in C1 rather than C2 or C3, although there was a globally increasing expression of MFGE8 with a reduction in BAMBI expression in all three clusters (Figure 3G and Figure S3H, Supporting Information). Thus, BAMBI^high^MFGE8^high^ cells represent the C1 cluster of heterogeneous UC‐MSCs from the perspective of the single‐cell transcriptome.

### 2.4 Characterization and Phenotyping of BAMBI^high^MFGE8^high^ C1 UC‐MSCs

**Figure 4:**
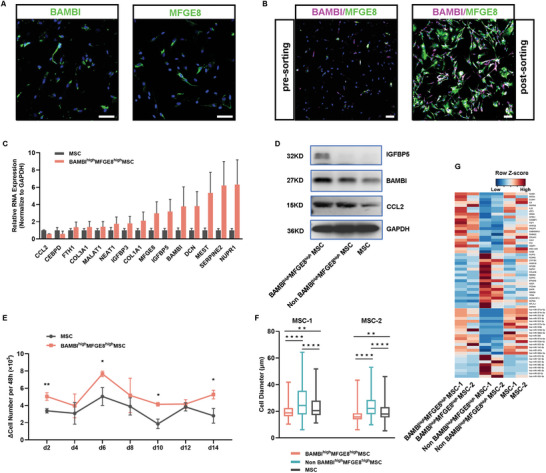
Isolation and characterization of phenotypic features of BAMBI^high^MFGE8^high^ C1 UC‐MSCs. A) BAMBI and MFGE8 expression in UC‐MSCs in culture. Scale bar: 50 µm. B) Pre‐ and post‐FACS sorting of BAMBI^high^MFGE8^high^ UC‐MSCs. Scale bar: 50 µm. C) The mRNA expression of signature genes of BAMBI^high^MFGE8^high^ UC‐MSCs examined by RT–qPCR. D) Representative western blotting of IGFBP5, CCL2 and BAMBI in BAMBI^high^MFGE8^high^, non‐BAMBI^high^MFGE8^high^ and unsorted UC‐MSCs. E) Growth curves of changes in cell number per 48 h for the BAMBI^high^MFGE8^high^ and unsorted UC‐MSCs in 14 days of expansion. *n* ≥ 3; Student's *t*‐test; *, *p* < 0.05, **, *p* < 0.01. F) Cell diameters of BAMBI^high^MFGE8^high^, non‐BAMBI^high^MFGE8^high^ and unsorted UC‐MSCs. *n* = 122‐281 for MSC‐1 and *n* = 182‐312 for MSC‐2; Student's *t‐*test; **, *p* < 0.01, ****, *p* < 0.0001. G) Heatmap of representative genes by mRNA, microRNA and lncRNA sequencing in BAMBI^high^MFGE8^high^, non‐BAMBI^high^MFGE8^high^, and unsorted UC‐MSCs.

To test the possibility of using BAMBI and MFGE8 for fluorescence‐activated cell sorting (FACS), we first verified their expression in UC‐MSCs by immunofluorescence. The results showed that both signals were heterogeneously located in UC‐MSCs with or without Triton X‐100 treatment (**Figure** **4**A and Figure S4A, Supporting Information). Similar signal patterns from intracellular and plasma membranes indicate their close positive correlation between cytoplasmic and transmembrane located BAMBI as well as MFGE8 (Figure S4A, Supporting Information). We then successfully pooled BAMBI^high^MFGE8^high^ UC‐MSCs by FACS from whole UC‐MSCs in culture (Figure 4B and Figure S4B, Supporting Information). Consistent with the single‐cell transcriptomic results, the enriched BAMBI^high^MFGE8^high^ UC‐MSCs showed higher expression of signature genes in C1 (NUPR1, SERPINE2, MEST, DCN, BAMBI, IGFBP5, MFGE8, COL1A1, IGFBP3, and NEAT1) by RT–qPCR (Figure 4C). The higher expression of IGFBP5, BAMBI, and CCL2 at the protein level was also validated by western blot (Figure 4D). Hence, the FACS‐sorted BMABI^high^MFGE8^high^ cells represent the C1 cluster in the UC‐MSCs.

To further characterize the BAMBI^high^MFGE8^high^ UC‐MSC phenotype, we tracked their growth conditions every 48 h for 14 consecutive days. Cellular growth curves showed that there were more BAMBI^high^MFGE8^high^ cells at each checkpoint until 14 days than their equivalent unsorted UC‐MSCs (Figure 4E and Figure S4C, Supporting Information), suggesting a faster proliferation rate for the BAMBI^high^MFGE8^high^ C1 subgroup. In fact, BAMBI^high^MFGE8^high^ C1 cells were significantly smaller and shorter by morphology in regular expansion than the other cells (Figure S4D, Supporting Information). In both UC‐MSC lines, the average diameter of BAMBI^high^MFGE8^high^ cells was significantly (*p* < 0.01) less than that of non‐BAMBI^high^MFGE8^high^ cells and unsorted MSCs (Figure 4F). Thus, the BAMBI^high^MFGE8^high^ C1 subpopulation is a distinct phenotypic cell population in heterogeneous UC‐MSCs.

To better understand the transcriptomic features of the C1 cluster, we performed further mRNA, microRNA, and lncRNA sequencing of BAMBI^high^MFGE8^high^ cells in culture (Figure 4G). The heatmap reveals upregulation of genes controlling cellular proliferation (FGF1, CGGBP1), cell stress (GABARAPL1, ATF3), matrix modeling (FN1, COL4A1, GABARAPL1, KLKB1), and inflammation and immune response (NFATC2, OAS2, GFRA1, IL32, IL6, KLKB1). A series of microRNA variances were also found between BAMBI^high^MFGE8^high^ and non‐BAMBI^high^MFGE8^high^ UC‐MSCs (Figure S5A,B, Supporting Information), in which many of them (hsa‐miR‐134‐5p,^[^ ^19^ ^]^ hsa‐miR206,^[^ ^20^ ^]^ hsa‐miR223,^[^ ^16^ ^]^ hsa‐miR‐519, etc.) may target the above genes and regulate mitotic cell division. Notably, hsa‐miR‐138‐5p and hsa‐miR‐4683 are important microRNAs for directing proliferation, lipid metabolism and the inflammatory response via other mRNAs and lncRNAs (Figure S5C,D, Supporting Information). Taken together, BAMBI^high^MFGE8^high^ cells are a unique subpopulation of UC‐MSCs.

### 2.5 Primed BAMBI^high^MFGE8^high^ C1 UC‐MSCs with Compromised Adipogenic Differentiation Potential

**Figure 5:**
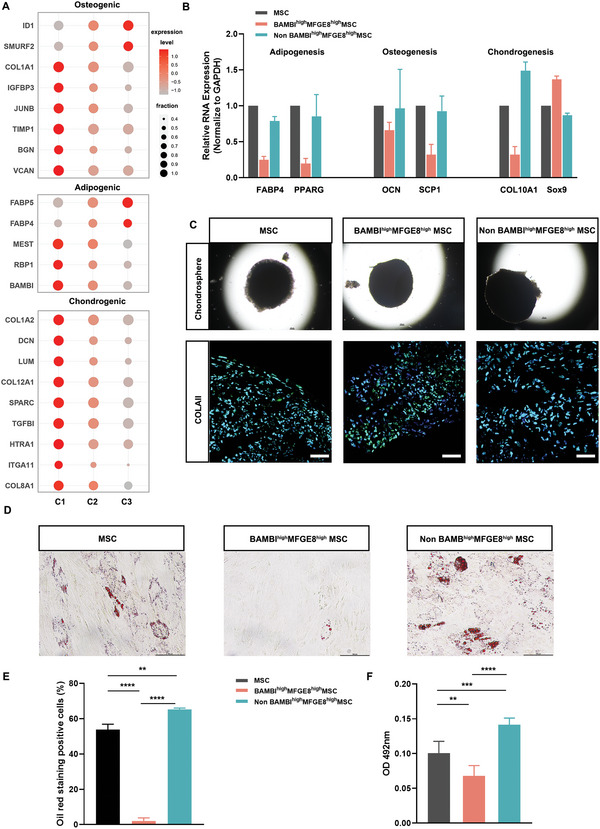
Primed osteochondrogenic BAMBI^high^MFGE8^high^ UC‐MSCs had compromised adipogenic differentiation potential. A) Bubble plot of highly expressed osteogenic, chondrogenic, and adipogenic marker genes in the C1 to C3 clusters in UC‐MSCs at P0. B) The expression of multiple differentiation markers of adipocytes (FABP4, PPARG), chondrocytes (COL10A1, Sox9) and osteoblasts (OCN, SCP1) of BAMBI^high^MFGE8^high^ UC‐MSCs, non‐BAMBI^high^MFGE8^high^ UC‐MSCs and unsorted UC‐MSCs examined by RT–qPCR following inductive differentiation. C) Chondrospheres and collagen II expression by immunofluorescence in BAMBI^high^MFGE8^high^ UC‐MSCs, non‐BAMBI^high^MFGE8^high^ UC‐MSCs, and unsorted UC‐MSCs. Scale bar: 100 µm. D) Oil red O staining of BAMBI^high^MFGE8^high^ UC‐MSCs, non‐BAMBI^high^MFGE8^high^ UC‐MSCs and unsorted UC‐MSCs after adipocytic differentiation treatment. E) Percentages of adipocytes positive for oil red O staining for BAMBI^high^MFGE8^high^ UC‐MSCs, non‐BAMBI^high^MFGE8^high^ UC‐MSCs and unsorted UC‐MSCs. *n* = 3 fields with 159–255 cells per field; Student's *t*‐test; **, *p* < 0.01, ****, *p* < 0.0001. F) Absorbance measurement of lipid accumulation extracted from BAMBI^high^MFGE8^high^ UC‐MSCs, non‐BAMBI^high^MFGE8^high^ UC‐MSCs, and unsorted UC‐MSCs. *n* = 3; Student's *t*‐test; **, *p* < 0.01, ****, *p* < 0.0001.

To determine whether the BAMBI^high^MFGE8^high^ cluster has its own special functional features within the mixed UC‐MSCs, we first tested its differentiation potential, as previous scRNA‐seq data suggest that the three groups are not in the same stem cell states (Figure 2). Further deeper examination of full osteogenic, chondrogenic and adipogenic markers used for clustering UC‐MSCs shows that there is higher expression of osteogenic markers (COL1A1, IGFBP3, JUNB, VCAN, etc.) and chondrogenic (COL1A2, DCN, LUM, TGFBI, etc.) genes in the C1 cluster than in the C2 or C3 cluster (**Figure** **5**A). For adipogenic differentiation, BAMBI^high^MFGE8^high^ C1 features higher expression of markers inhibiting adipogenesis (MEST, RBP1, and BAMBI). In contrast, there is higher expression of genes (FABP4 and FABP5) upregulating adipocyte differentiation in C3. Additionally, the C3 cluster also features higher expression of osteoblast precursor markers (ID1 and SNURF2). These results imply that BAMBI^high^MFGE8^high^ C1 cells are likely primed to be a chondro‐osteogenic lineage with limited adipogenic potential.

To this end, BAMBI^high^MFGE8^high^ cells were induced to differentiate into adipocytes, osteoblasts and chondrocytes under differentiation conditions, and the qPCR results indicated that there were no great expression changes for genes related to osteogenic (OCN, SCP1) and chondrogenic (COL10A1, Sox9) differentiation among BAMBI^high^MFGE8^high^ cells, non‐BAMBI^high^MFGE8^high^ cells and their unsorted populations (Figure 5B). Further immunofluorescence confirmed the qPCR results and demonstrated that the three groups of cells could differentiate into chondrocytes (Figure 5C). We next tested the adipocyte differentiation ability of the cells, and oil red O staining showed that only 2.01% of BAMBI^high^MFGE8^high^ cells differentiated into adipocytes in vitro while there were over 50% of differentiated adipocytes on average for the non‐BAMBI^high^MFGE8^high^ UC‐MSCs and UC‐MSC groups (Figure 5D,E). Further measurement of extracted lipid quantity from the differentiated cells confirmed that there was a significant difference regarding lipid production across the three UC‐MSC groups, in which over twofold less lipid absorbance value was detected from the BAMBI^high^MFGE8^high^ subpopulation than the non‐BAMBI^high^MFGE8^high^ UC‐MSCs (Figure 5F). Thus, BAMBI^high^MFGE8^high^ UC‐MSCs represent an MSC subtype with limited adipogenic potential.

### 2.6 BAMBI^high^MFGE8^high^ C1 UC‐MSCs Failed to Alleviate SLE in MRL/lpr Mice

**Figure 6:**
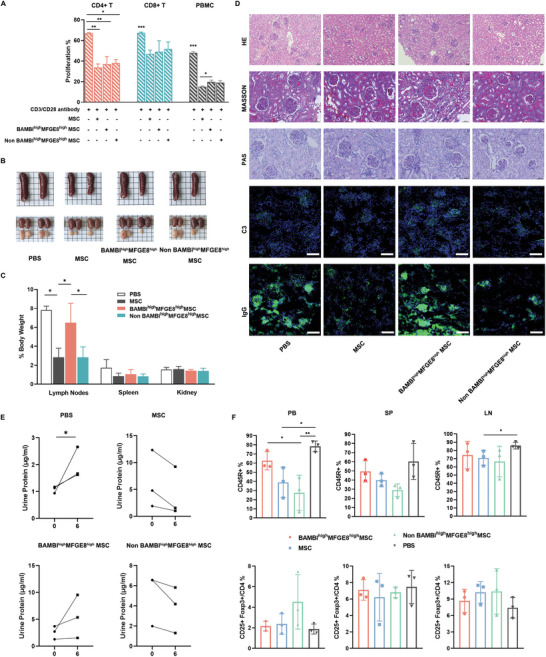
BAMBI^high^MFGE8^high^ UC‐MSC transplantation failed to alleviate SLE in MRL/lpr mice. A) Flow cytometry analysis of CD4^+^ T, CD8^+^ T‐cell and PBMC proliferation with BAMBI^high^MFGE8^high^ UC‐MSCs, non‐BAMBI^high^MFGE8^high^ UC‐MSCs or UC‐MSCs co‐cultured in vitro. B) Photos of spleens, kidneys, and renal lymph nodes dissected after MSC treatment (day 6) in MRL/*lpr* mice. C) Relative weights of the lymph nodes, spleen and kidney of MRL/*lpr* mice after treatment. *n* = 3; Student's *t*‐test; *, *p* < 0.05. D) Representative images of renal HE, Masson, and PAS sections and IgG and C3 deposits in the kidneys of MRL/*lpr* mice. Scale bar: 50 µm. E) Urinary albumin excretion curves and proteinuria at day 0 and day 6 after MSC treatment in MRL/lpr mice. F) Percentage of Treg cells in peripheral blood, spleen, lymph nodes and CD45R^+^(B220^+^) cells in the MRL/*lpr*. *n* = 3; one‐way ANOVA; *, *p* < 0.05; **, *p* < 0.01.

Allogenic transplantation of UC‐MSCs has been shown to alleviate SLE in both patients and MRL/lpr mice by suppressing overactivated immune cells and promoting Treg cells.^[^ ^3^, ^21^ ^]^ To understand whether the BAMBI^high^MFGE8^high^ subgroup could play excellent anti‐inflammatory roles in treating SLE, we first tested their suppressive function on T‐cell proliferation in a direct co‐culture system with PBMCs in vitro. The results showed that there was no significant difference in the BAMBI^high^MFGE8^high^ cells suppressing CD4^+^ and CD8^+^ T‐cell proliferation compared to the other two cell types, causing ≈30% and ≈20% reductions in CD4^+^ or CD8^+^ T cells, respectively (**Figure** **6**A). However, the BAMBI^high^MFGE8^high^ subtype is significantly less competent to inhibit PBMC proliferation (*p* < 0.05), implying their compromised ability to inhibit the expansion of other unknown cells in PBMCs.

We then transplanted BAMBI^high^MFGE8^high^ UC‐MSCs into MRL/lpr mice to test their therapeutic effects on SLE in vivo. Compared to the control group, both the UC‐MSC and non‐BAMBI^high^MFGE8^high^ UC‐MSC treatments led to apparently smaller renal lymph nodes and markedly reduced the total weight of lymph nodes, referred to as body weight, in the mice (Figure 6B,C). In contrast, BAMBI^high^MFGE8^high^ UC‐MSC transplantation did not show such beneficial effects. Accordingly, renal pathological section staining by hematoxylin and eosin (H&E), Masson and PAS revealed that BAMBI^high^MFGE8^high^ UC‐MSC treatment did not prevent lymphocyte infiltration, glycogen, collagen with myofibrosis formation, or IgG and C3 complement deposition. However, non‐BAMBI^high^MFGE8^high^ UC‐MSC and unsorted MSC transplantation alleviated renal pathological severity, especially IgG accumulation in glomeruli (Figure 6D). Further measurement of urine protein also verified that BAMBI^high^MFGE8^high^ UC‐MSC transplantation was incapable of preventing an increase in urinal protein, a classic marker of nephritis (Figure 6E). Finally, we detected immune cell subsets, including T, B, and Treg cells and etc. in peripheral blood, spleen and renal lymph nodes by flow cytometry and found that there was no significant difference in CD4^+^ T, CD8^+^ T, and Treg cell fractions among the control group and the 3 MSC‐treated groups, especially in PBMCs and LNs (Figure 6F and Figure S6, Supporting Information). However, similar to that of the PBS group, the ratio of CD45R^+^ B cells of the BMABI^high^MFGE8^high^ group was dramatically higher than that of the MSC or non‐BMABI^high^MFGE8^high^ group in the SLE mice (Figure 6F), possibly failing to suppress overactive Ig antibody production. Hence, BMABI^high^MFGE8^high^ UC‐MSCs represent a special subtype deficient in alleviating lupus nephritis, possibly because of their inability to properly adjust B‐cell function.

## 3 Discussion

A large‐scale human UC‐MSC single‐cell transcriptomic atlas was drawn by sequencing 12 samples of over 10^5^ cells from both early (P0) and late passages (P7) in this study. This new heterogeneity map shows a comprehensive profile of the cellular composition of human UC‐MSCs compared to those from primary studies on UC‐MSC heterogeneity based on limited samples.^[^ ^11^, ^12^, ^13^, ^22^ ^]^

Our drafted human UC‐MSC transcriptomic atlas provides increasingly deeper insight into UC‐MSC heterogeneity by uncovering not only the composition of different subgroups but also their stability in regular cell culture. Three distinct clusters (C1–C3) are inclined to be at a developmental primed (C1), intermediate (C2) or stemness (C3) status, which is further validated by examining their trilineage differentiation capabilities in vitro. Interestingly, the BAMBI^high^MFGE8^high^ C1 subgroup was proven to be hardly permissive for adipogenesis, which confirmed their poor stemness state in the heterogeneous population. Unexpectedly, human UC‐MSC heterogeneity was revealed to be relatively stable. The C1–C3 clusters contribute to all 12 scRNA‐sequenced samples with only limited variation in the C1, C2, and C3 fractions in individual donors. Only the expression of 6 genes (data not shown) was slightly but significantly changed in the C1–C3 subgroups from P0 to P7. Overall, ≈10% of C2 increases at the cost of C1 and C3, which may be attributed to the mutual interactions between the three subpopulations during cellular expansion in culture. As donor variation causes distinct therapeutic outcomes of human UC‐MSCs,^[^ ^21a^ ^]^ such stable heterogeneity strongly suggests that both competent and incompetent subtypes coexist in the mixed UC‐MSC population. Indeed, the BAMBI^high^MFGE8^high^ C1 subgroup failed to alleviate lupus nephritis in this study. However, the minimal percentage of C1 in the whole MSC population that will lead to failure of SLE treatment is not known at all, as depletion of the C1 subgroup from donor S1 covering 12.16% and 4.31% at the mRNA level at P0 and P7, respectively, did not seem to promote the beneficial effects of non‐C1 MSCs as examined in the lupus mice (Figure 6). Hence, it is worth comparing the therapeutic effects of UC‐MSCs from donors S1–S6 at P7 to determine the decisive fraction threshold.

Regardless of how many subgroups are classified for human heterogeneous UC‐MSCs by scRNA sequencing, it is essential to isolate those subtypes to further explore their characteristics and functions. However, it is still a major challenge to identify specific subgroups from all kinds of tissue‐derived MSCs, including UC‐MSCs. Neither conventional CD markers^[^ ^13^, ^14^ ^]^ nor recently proposed markers (e.g., CD142 and CD166/CD168)^[^ ^11^, ^12^, ^15^ ^]^ are suitable to separate C1–C3 subtypes simultaneously. For the first time, we successfully sorted the C1 cluster by using a new strategy of both BAMBI and MFGE8 transmembrane proteins, which eventually made it successful to experimentally authenticate the true uniqueness of the C1 subgroup within human UC‐MSCs regarding its smaller size, faster growth, different microRNA transcriptome profiles, incompetent adipogenic differentiation potential, and compromised immune suppressive function in terms of alleviating lupus nephritis. The characteristics of the BAMBI^high^MFGE8^high^ C1 cells are consistent with previous observations regarding morphological variations recorded by time‐lapse for single‐cell clones^[^ ^9^ ^]^ and inhibited adipogenesis regulated by BAMBI and MEST in MSCs.^[^ ^17^, ^23^ ^]^ Although it is not known how BAMBI and MFGE8 interact to inhibit adipogenic differentiation and attenuate immunoregulatory activity simultaneously in C1 cells, BAMBI is assumed to play a vital role, as it is also involved in commanding Th17/Treg differentiation.^[^ ^17^ ^]^ Therefore, we disclosed the unique features of the BAMBI^high^MFGE8^high^ C1 subgroup hidden in the heterogeneous UC‐MSC population.

To completely uncover the mystery of UC‐MSC heterogeneity, more work needs to be done due to the limitations of this study. For instance, the functions of C2 and C3 are not explored apart from the BAMBI^high^MFGE8^high^ C1 subgroup. Further isolation of the BAMBI^low^MFGE8^−^ C2 and BAMBI^−^MFGE8^−^ C3 subtypes is expected to clarify their real functions. Even for the C1 subgroup, extensive studies need to be performed to uncover its full functions beyond adipogenesis and immunomodulation in SLE. In particular, whether BAMBI^high^MFGE8^high^ C1 cells can still play a strong anti‐inflammatory role remains to be addressed in other experimental contexts and disease models. In addition, the interactions between C1–C3 have not been dissected experimentally. It is not clear whether application of a single subpopulation would achieve better therapeutic effects on treating diseases or combinational ones of C1–C3. Moreover, it is important to further dissect the signaling pathways and develop optimal culture conditions to maintain C1–C3 properties in the long term for future cell therapy applications because UC‐MSCs are concerned with losing their original properties regarding gene expression patterns and activities under inappropriate culture conditions in vitro.^[^ ^24^ ^]^

## 4 Conclusion

In the present study, we constructed a new comprehensive human UC‐MSC atlas by using large‐scale single‐cell transcriptomes, in which UC‐MSC heterogeneity was dissected to contain three subgroups (C1–C3) with their own signatures. More importantly, the pooled BAMBI^high^MFGE8^high^ C1 UC‐MSCs were further phenotypically and functionally characterized to be unique with incompetent adipogenic differentiation potential and compromised immunosuppressive activity in alleviating lupus in mice. This study is helpful to clarify the nature of UC‐MSCs and improve their clinical efficacy by choosing optimal types of MSCs to treat specific diseases in the future.

## 5 Experimental Section

### Isolation and Culture of UC‐MSCs

This study was conducted in accordance with the principles set forth under the 1989 Declaration of Helsinki and approved by the Ethics Committee at the Affiliated Drum Tower Hospital of Nanjing University Medical School (approval number: 202019701). Human umbilical cords were obtained from informed healthy mothers in The Affiliated Drum Tower Hospital of Nanjing University Medical School after natural labor. The umbilical cords were transferred to phosphate‐buffered saline (PBS) supplemented with 200 U mL^−1^ penicillin and streptomycin.

Human umbilical cords were collected and cut into 2–3 cm^3^ pieces in RPMI 1640 medium with 100 U mL^−1^ penicillin and 100 µg mL^−1^ streptomycin. After removing two arteries and one vein from the umbilical cord piece using forceps and a scalpel holder, the umbilical cord was chopped into smaller 3–5 mm^3^ pieces, fresh complete culture medium was added, and the dishes were placed in a CO_2_ incubator maintained at 37 °C and 5% CO_2_. After 48 h, the fresh culture medium was changed in the dishes. Then, media changes were given every 72 h. Once the MSCs were 70–80% confluent, the cells of each dish were transferred to three dishes with trypsin (TrypLE Express, 12604021, Thermo Fisher).

After two passages, the cells were harvested and characterized for MSC surface markers with flow cytometry. Flow cytometric analysis confirmed the expression (≥95%) of CD73, CD105, CD90, and CD29 and the absence (≤2%) of CD45, CD34, CD14, CD79, and HLA‐DR on these cells. The trilineage differentiation capacity of MSCs during adipogenesis and osteogenesis and chondrogenesis was also assayed and confirmed.

### Cell Growth Assay and Size Measurement

To measure the size of UC‐MSCs, before each passage expansion, the cells were split into single cells by 0.25% trypsin‐EDTA, stopping digestion by MSC complete medium. Ten microliters of the cell suspension was added to the cell counter, and photos of three to four fields were taken at random with NIS‐Elements F software by a microscope (Nikon, Japan). All pictures were applied to Nano Measure 1.2.5v software to measure the cell sizes.

For the proliferation assay, 2.5 × 10^5^ cells were seeded into three T25 flasks for each cell line at each passage with MSC complete medium. 48 h later, the cells were digested with 0.25% trypsin‐EDTA and counted to record the cell numbers.

### Differentiation of MSCs

According to the manufacturer's protocols, direct differentiation of human UC‐MSCs toward adipocytes, osteoblasts, and chondrocytes was initiated with a Human MesenCult Adipogenic Differentiation Kit (05412, STEMCELL Technologies Inc., Canada), Human MesenCult Osteogenic Differentiation Kit (05465, STEMCELL Technologies Inc., Canada), and MesenCult‐ACF Chondrogenic Differentiation Kit (05455, STEMCELL Technologies Inc., Canada), respectively. Two to four weeks later, the cells were either fixed for Oil Red (O1391, Sigma–Aldrich, USA) staining and immunostaining or exacted by using RNA isolate Total RNA Extraction Reagent (R401‐01, Vazyme, China) for RNAs. To calculate the differentiation efficiency of adipocytes, photos of three fields at random were taken with a 10× objective lens with NIS‐Elements F software by a microscope (Nikon Ts2‐FL, Japan) for the UC‐MSCs after Oil Red O staining. Then the cells were counted with the ImageJ 1.52v software for further analysis. For quantification of lipids from oil red‐stained cells, 100% isopropanol was used to extract oil red for 5 min with gentle rocking. The absorbance at 492 nm was read by a Synergy HT Multi Detection Reader (BioTek Instruments, Winooski, VT) for the extracted liquid.

### Flow Cytometry and Fluorescence Activated Cell Sorting

The murine spleen and perinephric renal lymph node were ground and passed through 70 µm cell strainers twice. Harvested single cells were suspended in FACS buffer (PBS supplemented with 2% FBS, 1 mM EDTA, and 1% penicillin–streptomycin). Then, peripheral blood and single cells from the spleen and perinephric renal lymph nodes were lysed in ACK buffer (0.15 M NH_4_Cl, 10 mM KHCO_3_, 0.5 mM Na_2_EDTA, pH 8.0) to remove red cells. After washing twice with FACS buffer (PBS with 1% BSA), the cells were stained with Fixable Viability Dye eFluor 780 and relevant antibodies in FACS buffer for 30 min on ice. For intracellular staining, the cells were prepared by a transcription factor buffer set (51‐9008102, BD Biosciences). Cells were stained with the antibodies described in Table S3, Supporting Information. Flow cytometric analysis was processed by a Fortessa (Becton Dickinson), and the data were analyzed with FlowJo software.

To prepare UC‐MSCs for cell sorting, UC‐MSCs were rinsed with PBS and digested with 0.5 mM EDTA for 3 min. Complete MSC culture medium was added, and the cell suspension was further pipetted completely before filtering with a 70 µm cell strainer to collect single cells. The cells were centrifuged at 300 × *g* for 5 min and then resuspended at 2 × 10^6^ cells per mL in the cell culture medium for appropriate antibodies. Mouse anti‐human MFGE8 antibody (1:200, ob388429, Biorbyt, UK) was added to the cell suspension and incubated for 15 min at room temperature. After centrifugation, the supernatant was removed, and the cell pellets were resuspended in the same volume of cell culture medium supplemented with both anti‐human BAMBI conjugated with Cy5 antibody (1:200, bs‐12418R‐Cy5, Bioss Antibodies, USA) and goat anti‐mouse Alexa 488 secondary antibody (1:1000, 4408S, CST). The cells were further incubated with the above antibodies for another 15 min before centrifugation to collect the cell pellets. The supernatant was removed, and PBS was added to wash the cells once before the cells were further used for cell sorting by an Arial III FACS machine (Becton Dickinson).

### PBMC Proliferation Inhibition Assay

MSCs (1 × 10^4^) were plated in 48‐well plates in DF12 (C11330500BT, Thermo Fisher Scientific) with 10% fetal calf serum (10099141C, Thermo Fisher Scientific) and 1% penicillin–streptomycin (SV30010, Cytiva) for overnight culture. PBMCs were isolated from human peripheral blood using the Ficoll–Paque method, labeled with eBioscience Cell Proliferation Dye eFluor 450 (65‐0842‐90, Thermo Fisher Scientific, USA), and plated at 1 × 10^5^ cells per well in the same plate treated with the presence of 1 µg mL^−1^ anti‐CD3 (16‐0037‐81, Thermo Fisher Scientific, USA) and anti‐CD28 (16‐0289‐81, Thermo Fisher Scientific, USA). After four days of co‐culture, cells in suspension were collected by gently washing with PBS and then labeled with antibodies against CD4 (PE/Dazzle 594 anti‐human CD4, 562989, Biolegend) and CD8 (PerCP‐Cy5.5 Mouse Anti‐Human CD8, 560662, BD Biosciences) for flow cytometric analysis.

### Western Blotting

Cells were digested with trypsin (with 0.25% EDTA) to collect the cell pellets. RIPA buffer was used to lyse cell pellets. A BCA protein assay kit was used to measure the sample concentration and then normalize them. The samples were separated on a 10% SDS–PAGE gel (or 12.5% SDS–PAGE gel) and transferred to PVDF membranes. After blocking with 5% BSA for 1 h at room temperature, the membranes were incubated with anti‐IGFBP5 (1:1000, sc‐515184, Santa Cruz, USA), anti‐BAMBI (1:500, bs‐12418R, Bioss Antibodies, USA), anti‐CCL2 (1:500, 66272‐1‐Ig, Proteintech, China), and GAPDH (1:5000, 60004‐1‐Ig, Proteintech, China) primary antibodies at 4 °C overnight. After washing with TBST, the PVDF membranes with immunocomplexes were incubated with horseradish peroxidase‐conjugated anti‐rabbit or anti‐mouse IgG (Fcmacs) secondary antibodies at room temperature for 30 min. After washing with TBST, the PVDF membranes were detected with Super‐Signal West Pico PLUS (34577, Thermo Fisher Scientific).

### Quantitative RT–PCR

MSCs and sorted subtype MSCs were treated with Tri Reagent (T9424, Sigma) for total RNA extraction by phenol–chloroform. One microgram of RNA was reverse transcribed using HiScript II Q RT SuperMix for qPCR (R223, Vazyme) in a final volume of 20 µL. Each cDNA sample was amplified and quantified by real‐time PCR using an AceQqPCR SYBR Green Master Mix Kit (Q131, Vazyme). The relative expression levels of target genes were calculated using the 2^−ΔΔCT^ method normalized to GAPDH. All relevant primers are listed in Table S4, Supporting Information.

### Animals and Histological Analysis

MRL/Mpj‐Faslpr/J mice (000485) were obtained from Jackon Laboratories (USA). All mice used for experiments were aged 17 weeks and housed in a specific pathogen‐free environment at the animal center of the Affiliated Drum Tower Hospital of Nanjing University Medical School. All animal studies were performed according to the institutional guidelines of the Affiliated Drum Tower Hospital of Nanjing University Medical School and were approved by the Committee of Experimental Animal Administration of the Affiliated Drum Tower Hospital of Nanjing University Medical School (approval number: 2019AE01033). MRL/lpr mice were treated with 0.5–1 × 10^6^ UC‐MSCs or subtype MSCs via tail vein injection. All mice were sacrificed one week after MSC injection. Kidneys were collected for immunofluorescence and histological analysis.

For histological analysis, the harvested kidneys were fixed with 4% paraformaldehyde (PFA), embedded in paraffin, sectioned at 5 µm and stained with H&E, periodic acid‐Schiff (PAS), and Masson's trichrome stain.

Urine samples were collected before and after MSC injection. The concentrations of urinary protein were measured using a Bradford protein quantitation assay (KeyGEN, Nanjing, China).

### Immunofluorescence

Cells were fixed with 4% PFA in PBS for 30 min and permeabilized with (or without if needed) 0.4% Triton X‐100 (Sigma) for 10 min. Nonspecific binding sites were blocked with 10% goat serum for 30 min at room temperature. Cells were then incubated overnight at 4 °C or 1 h at room temperature with anti‐BAMBI or anti‐MFGE8 primary antibodies (Table S3, Supporting Information). After three rinses in PBS, cells were exposed to goat anti‐mouse, anti‐rabbit immunoglobulin G conjugated either to Alexa Fluor 488 or 647 (1:1000, CST) for 1 h at room temperature, followed by nuclear staining with 5 µg mL^−1^ Hoechst 33258 for 10 min. After three rinses in PBS, coverslips were mounted on slides. The cells on coverslips were examined using a confocal laser scanning system (FV3000, Nikon, Japan). A similar procedure was used to immunostain the 10‐µm‐thick cryosections of MSC‐derived chondrospheres and the harvested kidneys against collagen II, C3 and IgG (Table S3, Supporting Information).

### Library Preparation and Sequencing

Single‐cell RNA‐seq was performed using the 10× Genomics Chromium Single Cell Controller with the Chromium Single Cell 3′ V2 Kit (Chromium Single Cell 3ʹ Library and Gel Bead Kit v2, 16 rxns PN‐120237, Chromium Single Cell A Chip Kit, 48 rxns PN‐120236, Chromium i7 Multiplex Kit and 96 rxns PN‐120262, all from 10× Genomics, USA) following the manufacturer's instructions with barcoded gel beads at a target capture rate of 10 000 individual cells per sample. After quality control, libraries were sequenced on the Illumina HiSeq X‐ten platform in 2 × 150 bp paired‐end mode.

### Quality Control and Processing of Single‐Cell RNA‐Seq Data

Raw sequencing data were processed with Cell Ranger v3.0.2 software (10× Genomics) for demultiplexing and alignment to the GRCh38 human reference transcriptome. Processed data were analyzed using R statistics software and various Bioconductor packages. Specifically, Seurat v3.1.2 was used to load preprocessed results from Cell Ranger into R v3.6.2 and to perform quality control (i.e., removing any cells meeting the following criteria: <2000 or >6000 unique genes expressed, <2000 or >50 000 UMIs, or >5% mitochondrial content).

To maintain a standard procedure for clustering, a value of 0.6 was used for the resolution. To investigate transcriptional heterogeneity and to undertake initial cell clustering, dimensionality reduction was applied with principal component analysis (PCA). Top 20 principal components (PCs) was selected that explained more variability than expected by chance using a permutation‐based test in Seurat. For cell clustering within primary clusters (subclustering), top 500 variable genes were selected for dimensionality reduction using either permutation‐based analyses or heuristic methods in Seurat. PC loadings were used as input for a graph‐based approach to cluster cells by cell type and as input for uniform manifold approximation and projection (UMAP) for reduction to two dimensions for visualization purposes. Cluster‐specific genes were acquired using the FindMarkers algorithm in the Seurat suite.

### Gene Set Enrichment Analysis

Each GO biological process term or KEGG pathway term was defined as a gene set to be imported into the Java Gene Set Enrichment Analysis (GSEA; version 4.1.0) platform for downstream analysis. The ranked list and permutation type of the gene set were generated based on the average gene levels of C1 and C3. Gene sets with a *p* value less than 0.05 and FDR value less than 0.25 were considered statistically significant.

### Cell Communication Analysis

To investigate potential interactions between C1 and C3, cell–cell communication analysis was performed using CellCall, an open‐source R package (https://github.com/ShellyCoder/cellcall). According to prior knowledge of L‐R‐TF interactions based on KEGG pathways, CellCall inferred intercellular communication by combining the expression of ligands/receptors and downstream TF activities for certain L‐R pairs.

### lncRNA Sequencing

An RNA library for lncRNA‐seq was prepared using the rRNA depletion and stranded method. Briefly, ribosomal RNA was depleted from total RNA using the rRNA Removal Kit following the manufacturer's instructions. RNA was then fragmented into 250–300 bp fragments and reverse‐transcribed to cDNA, which was followed by cDNA amplification using PCR. After library preparation and quality control, the samples were subjected to Illumina sequencing. RNA‐seq was performed using PE150 (paired‐end 150 nt) sequencing for 12G raw data.

Reads for each sample were first mapped to a reference genome with HISAT2 software. Read alignment results were transferred to the program StringTie for transcript assembly. EdgeR was used for differential expression analysis. The resulting *p* values were adjusted using Benjamini and Hochberg's approach for controlling the false discovery rate. Genes with |log2 (fold change) | > 0 and padj < 0.05 were considered differentially expressed.

### Small RNA‐Seq Assay

The small RNA libraries were prepared using NEBNext Multiplex Small RNA Library Prep Set for Illumina (NEB, USA.) according to the manufacturer's instructions. Then, PCR amplification was performed, and the clustering of the index‐coded samples was performed on a cBot Cluster Generation System using TruSeq SR Cluster Kit v3‐cBot‐HS (Illumina) according to the manufacturer's instructions. After cluster generation, the library preparations were sequenced on a NovaSeq 6000 platform, and 50 bp single‐end reads were generated.

Processed reads of length 18 to 35 nt were then mapped to their reference genome and analyzed using the bowtie package (no mismatch). To identify conserved miRNAs, the predicted miRNA hairpins were compared against miRNA precursor sequences from miRbase22.0.

### Statistical Analysis

All data were shown as the mean ± standard derivation (SD) of the mean. GraphPad Prime 8 software (GraphPad, USA) was used to analyze the data by Student's *t*‐test or one‐way ANOVA. A *p* value < 0.05 was considered statistically significant.

## Conflict of Interest

The authors declare no conflict of interest.

## Author Contributions

H.C., X.W., and S.L. contributed equally to this work. H.C., J.Y., and L.S. conceived the study; H.C. and S.L designed, performed, and analyzed the experiments; H.C., X.W., and S.L. prepared the figures; T.S., H.S., Y.Z., and Y.Z. aided in performing experiments; S.L. and X.W. contributed to scRNA‐seq cellular capture, library preparation, and sequencing; X.W. performed all scRNA‐seq computational analyses with advice and/or direction from H.C., F.W., and J.X. under the supervision of J.Y.; J.Y. and L.S. supervised the study; H.C. wrote the manuscript, which was edited by all co‐authors. L.S. provided all funding support. All authors have finally approved the manuscript.

## Supporting information

Supporting Information

Supporting Information

Supporting Information

Supporting Information

## Data Availability

The data that support the findings of this study are available in the Supporting Information of this article.
