# Supplementary material for: Dissecting Heterogeneity Reveals a Unique BAMBIhighMFGE8high Subpopulation of Human UC‐MSCs
Source: Adv Sci (Weinh). 2022 Nov 14;10(1):2202510. doi: 10.1002/advs.202202510 (PMC9811468; doi:10.1002/advs.202202510)
Supplement: Supplementary file 1 — Supporting Information [file ADVS-10-2202510-s002.pdf]

ADVANCED SCIENCE
Open Access

Supporting Information

for *Adv. Sci.*, DOI 10.1002/advs.202202510

Dissecting Heterogeneity Reveals a Unique BAMBI^{high}MFGE8^{high} Subpopulation of Human UC-MSCs

Hongwei Chen, Xin Wen, Shanshan Liu, Tian Sun, Hua Song, Fang Wang, Jiayue Xu, Yueyang Zhang, Yuanjin Zhao, Jia Yu and Lingyun Sun**

Supporting Information

Dissecting Heterogeneity Reveals a Unique BAMBI^{high}MFGE8^{high} Subpopulation of Human UC-MSCs

Hongwei Chen[+], Xin Wen[+], Shanshan Liu[+], Tian Sun, Hua Song, Fang Wang, Jiayue Xu, Yueyang Zhang, Yuanjin Zhao, Jia Yu, and Lingyun Sun**

This PDF file includes:

Figs. S1 to S6
Tables. S1 to S4
Legends for Data files S1 to S3

Other Supplementary Materials for this manuscript include the following:

Data files S1 to S3

Supplementary Figures

Figure. S1.

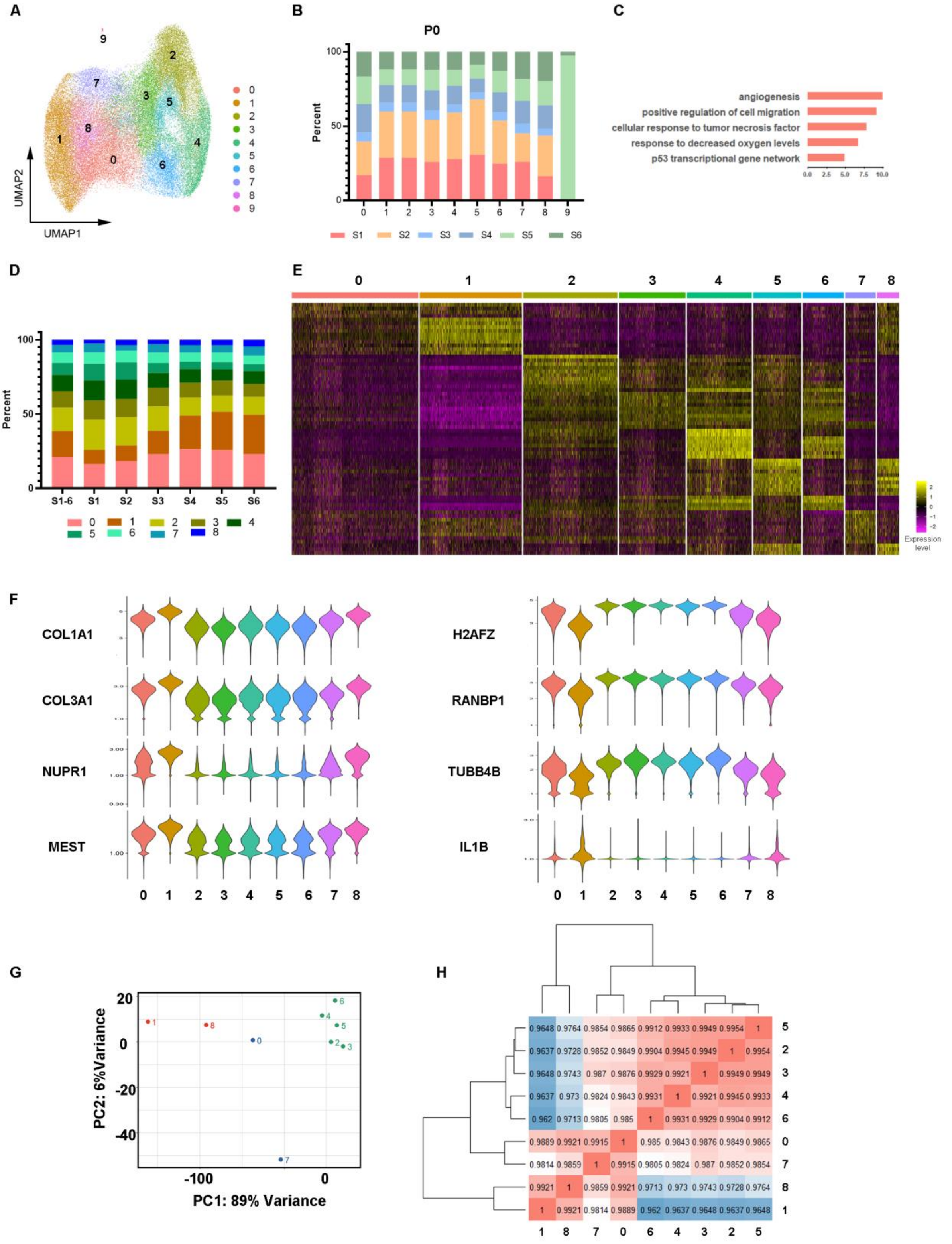

Supplementary Figure S1. UC-MSCs at P0 were a heterogeneous population. (**A**) UMAP visualization of single cell clusters of UC-MSCs at P0. (B) Distribution of the 6 donors among 10 subgroups at P0. (C) Enriched

pathways of subgroup 9 in UC-MSCs at P0. (D) Distribution of the 9 subgroups among 6 donors at P0. (E) Heatmap showing the expression levels of the top 10 differentially expressed genes in subgroups of UC-MSCs at P0. (F) Violin plot showing the expression of certain significant genes similar in some UC-MSC subgroups at P0. (G) Principal component analysis of all the clusters of UC-MSCs at P0. (H) Correlation analysis of UC-MSC subgroups at P0.

Figure S2

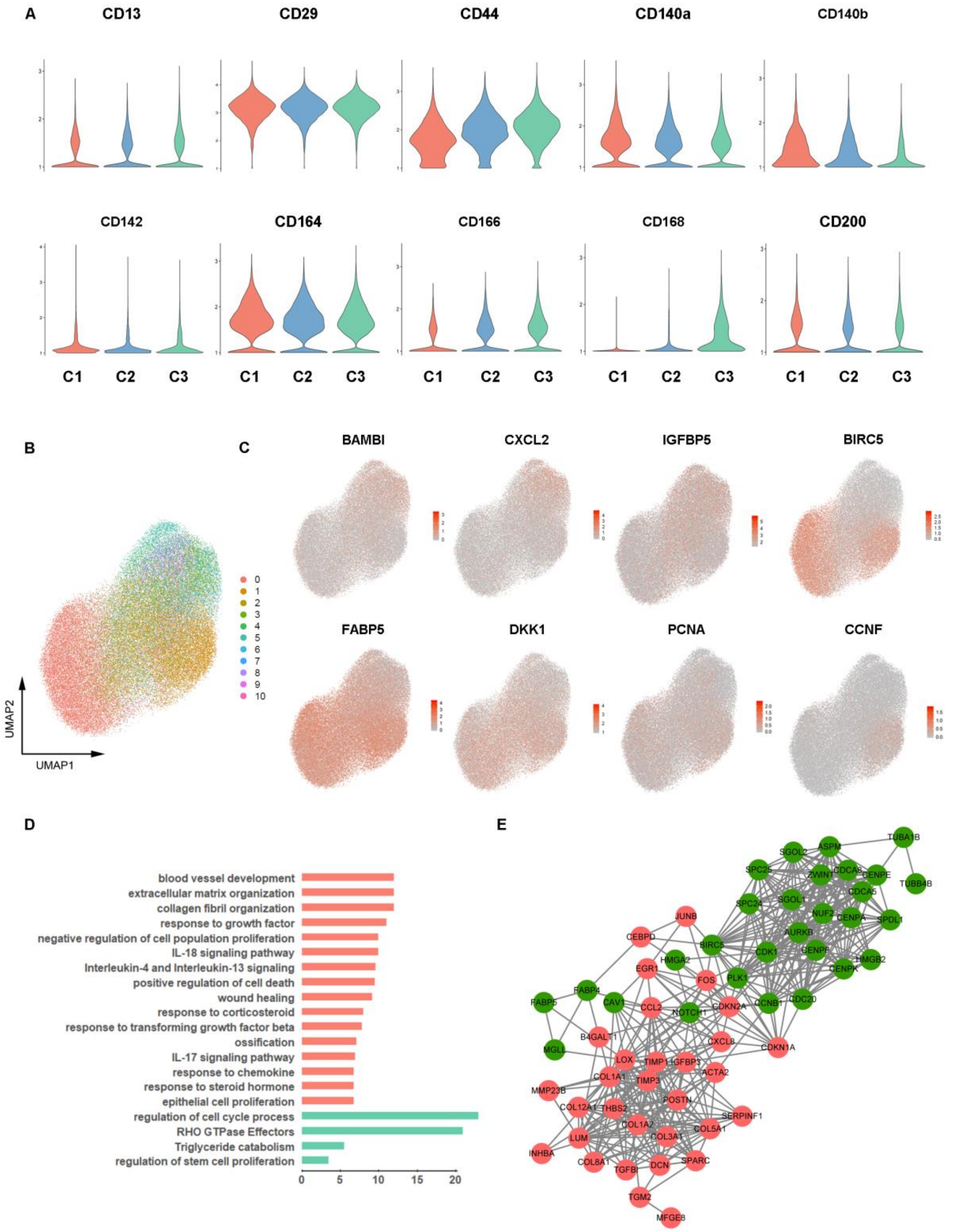

Supplementary Figure S2. Highly expressed genes and enriched pathways of P0 UC-MSC subgroups

(A) Violin plots showing limited known marker genes in P0 UC-MSC clustering. (B) UMAP visualization of single cell clusters of UC-MSCs at P0 by UMAP with cell cycle regression. (C) Feature plot showing gene expression levels in P0 UC-MSCs with cell cycle regression. (D) Enrichment pathway based on highly expressed genes of C1 and C3 (red represents the C1 enriched pathway and green represents the C3 enriched pathway). (E) Protein interaction network of C1 and C3 (red represents C1 signature genes, and green represents C3 signature

genes).

Figure. S3.

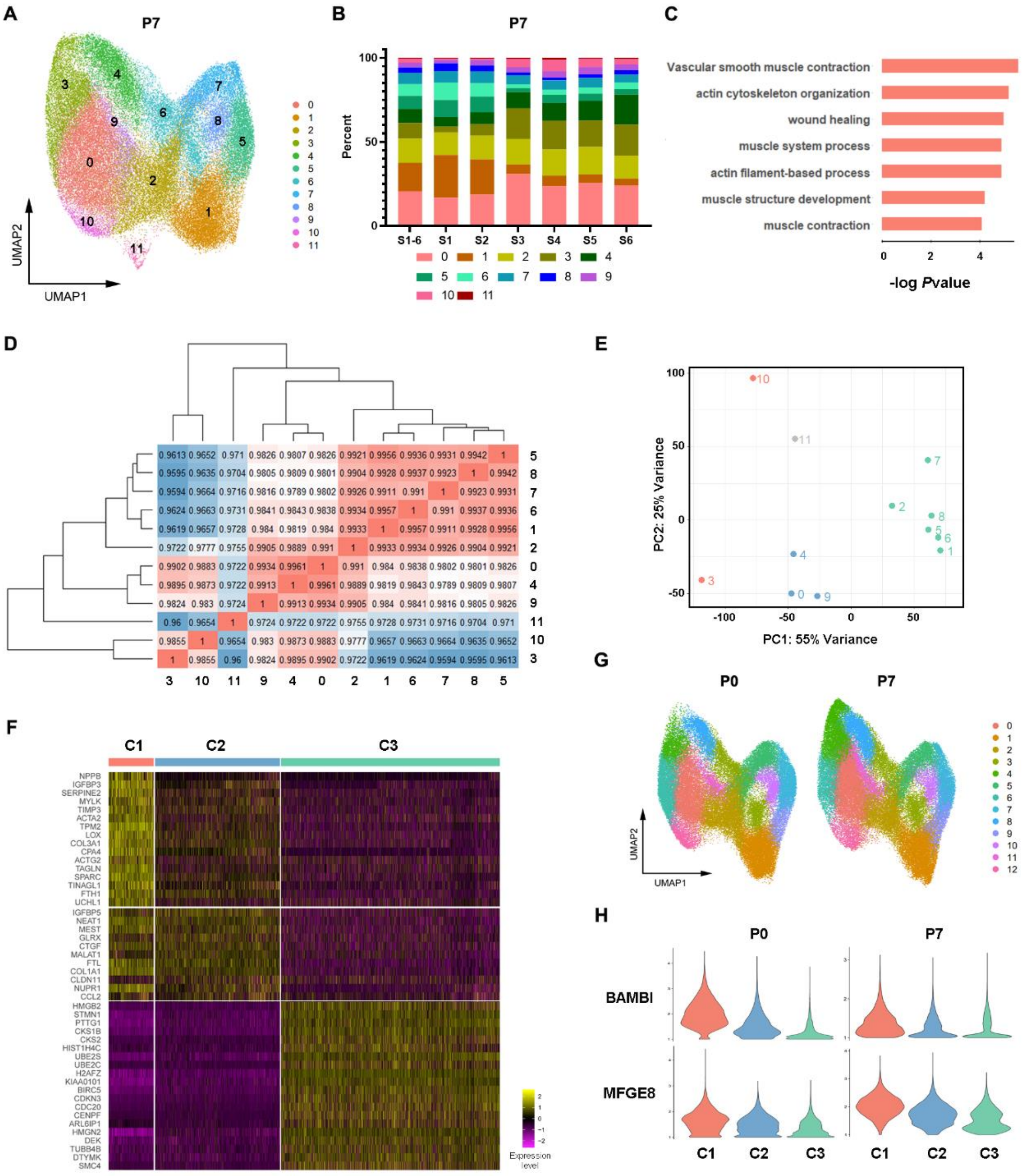

Supplementary Figure S3. The single-cell transcriptome of UC-MSCs at P7 showed a similar gene expression pattern to that at P0. (**A**) UMAP visualization of single cell clusters of UC-MSCs at P7. (B) Distribution of the 12 subgroups among 6 donors at P7. (C) Enriched pathways of subgroup 11 in UC-MSCs at P7. (D) Correlation analysis of P7 subgroups. (E) Principal component analysis of all the clusters of UC-MSCs at P7. (F) Heatmap showing the expression levels of the top 20 differentially expressed genes in subgroups of UC-MSCs at P7. (G) UMAP visualization of single cell clusters in UC-MSCs at P0 and P7. (H) Violin plots showing different expression levels of BAMBI and MFGE8 in the 3 subgroups of UC-MSCs at P0 and P7.

Figure. S4.

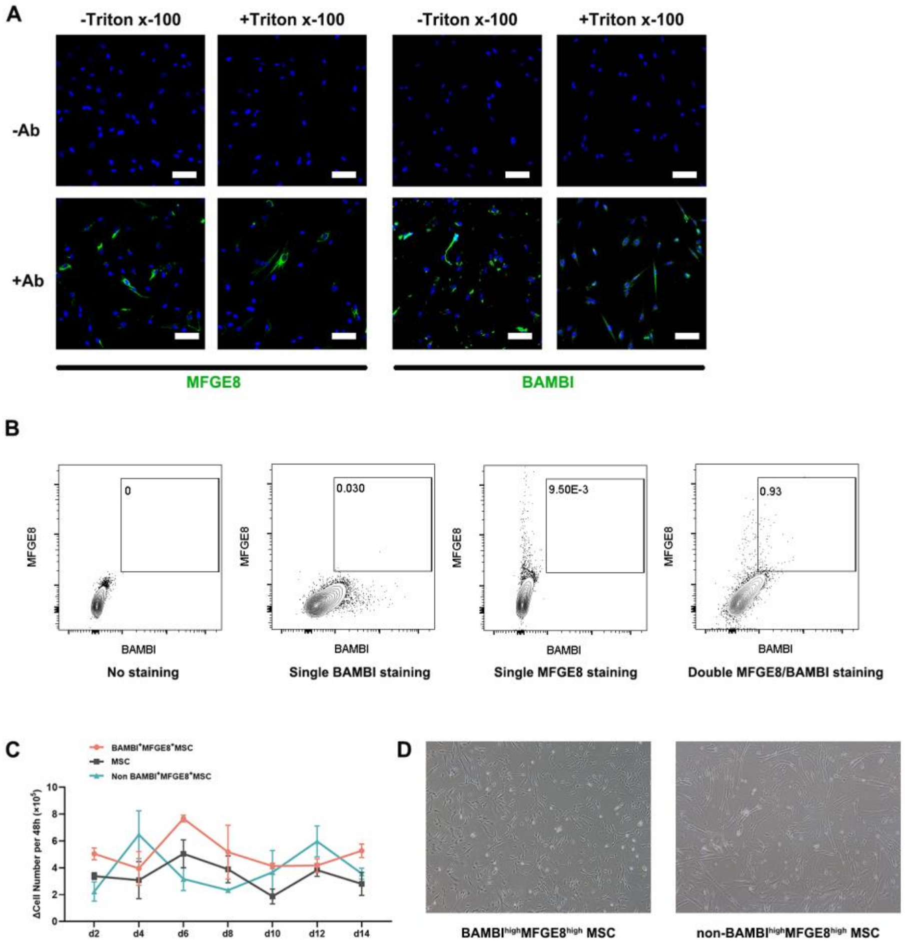

Supplementary Figure S4. Isolation and phenotyping of BAMBI^{high}MFGE8^{high} UC-MSCs. (**A**) BAMBI and MFGE8 immunostaining in UC-MSCs with and without Triton X-100. Scale bar: 50 μm. (B) Representative FACS sorting strategy of BAMBI^{high}MFGE8^{high} C1 UC-MSCs from UC-MSCs. (C) Growth curves of cell number changes per 48 hours for BAMBI^{high}MFGE8^{high}, non-BAMBI^{high}MFGE8^{high} and unsorted UC-MSCs; n ⩾3. (D) BAMBI^{high}MFGE8^{high} UC-MSCs and non-BAMBI^{high}MFGE8^{high} UC-MSCs in culture. Magnification: x40.

Figure. S5.

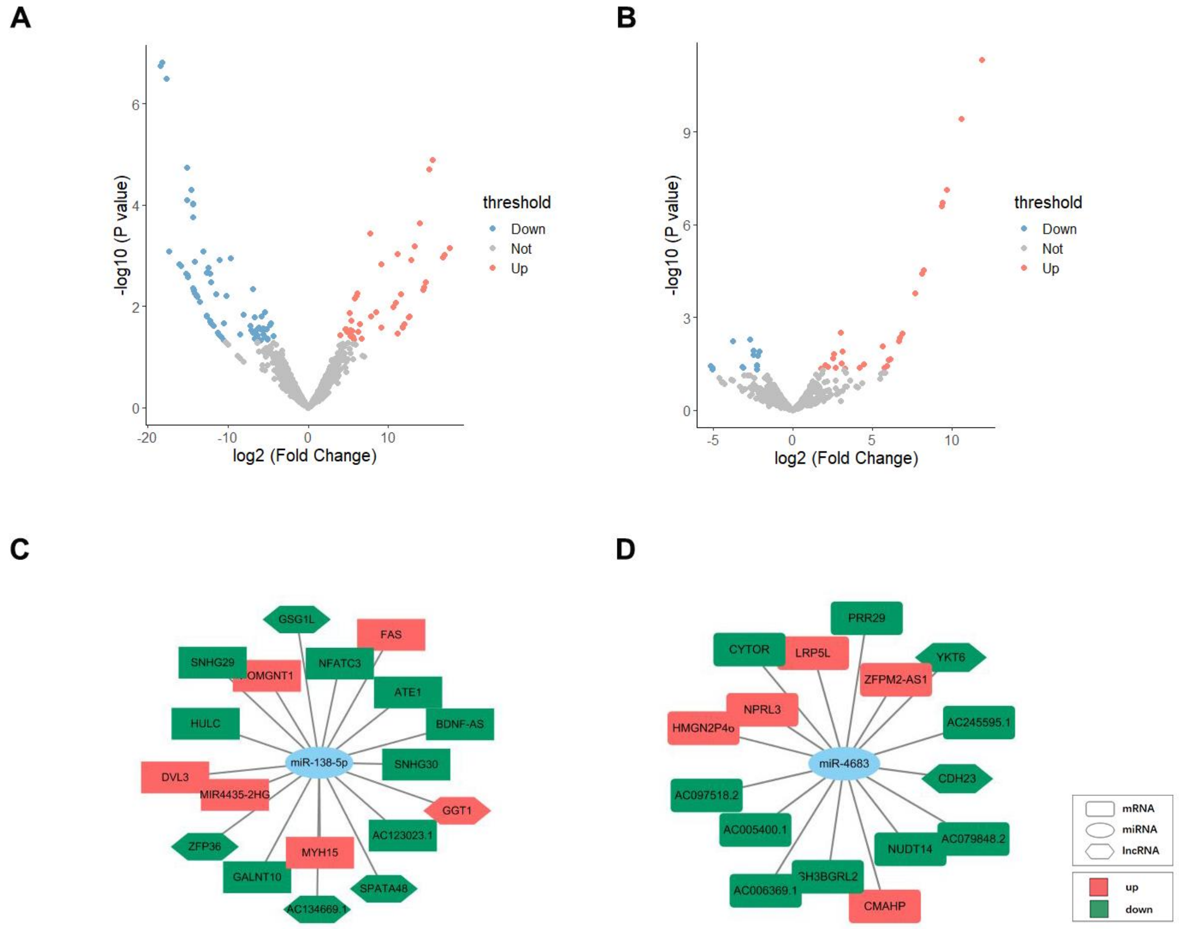

Supplementary Figure S5. mRNA, microRNA and lncRNA in UC-MSC subtypes. (**A**) Volcano plot of differentially expressed lncRNAs in BAMBI^{high}MFGE8^{high} UC-MSCs and non-BAMBI^{high}MFGE8^{high} UC-MSCs. (B) Volcano plot of differentially expressed microRNAs in BAMBI^{high}MFGE8^{high} UC-MSCs and non-BAMBI^{high}MFGE8^{high} UC-MSCs. (C) MiRNA-lncRNA-mRNA network of hsa-miR-138-5p in BAMBI^{high}MFGE8^{high} UC-MSCs vs unsorted UC-MSCs by WTS analysis. (D) MiRNA-lncRNA-mRNA network of hsa-miR-4683 in BAMBI^{high}MFGE8^{high} UC-MSCs vs unsorted UC-MSCs by WTS analysis.

Figure. S6.

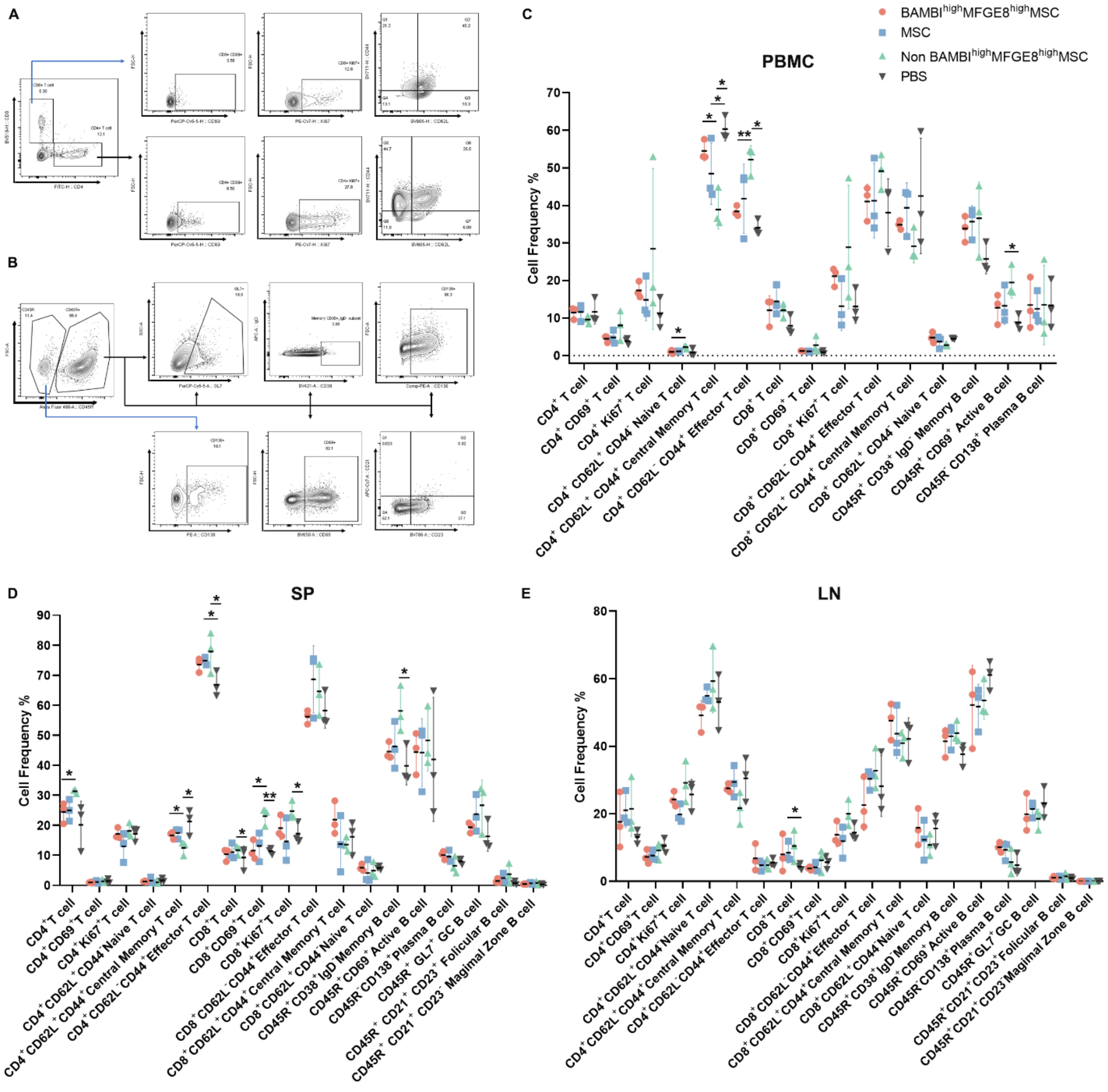

Supplementary Figure S6. Representative flow cytometry plots related to Fig 6. (**A**) Representative gating strategy for T-cell analysis in peripheral blood, spleen and lymph nodes. (B) Representative gating strategy for B-cell analysis in peripheral blood, spleen and lymph nodes. (C) $CD4^+$ T cells, $CD8^+$ T cells, B cells and the subsets were analyzed via flow cytometry from peripheral blood of MRL/*lpr* mice. (D) $CD4^+$ T cells, $CD8^+$ T cells, B cells and the subsets were analyzed via flow cytometry from the spleens of MRL/*lpr* mice. (E) $CD4^+$ T cells, $CD8^+$ T cells, B cells and the subsets were analyzed via flow cytometry from the perirenal lymph nodes of MRL/*lpr* mice. n=3; Student's *t*-test was used to determine significance; * indicates p<0.05. ** indicates p<0.01.

Supplementary Tables

Table S1.

Sample		*Gender*	*Passage*
S1	S1-P0	Female	0
	S1-P7		7
S2	S2-P0	Male	0
	S2-P7		7
S3	S3-P0	Male	0
	S3-P7		7
S4	S4-P0	Female	0
	S4-P7		7
S5	S5-P0	Female	0
	S5-P7		7
S6	S6-P0	Male	0
	S6-P7		7

Supplementary Table S1. General information of the umbilical cord donors and cell samples collected for scRNA-seq.

Table S2.

Sample	*Number of Cells*	*Mean Reads per Cell*	*Median Genes per Cell*	*Total genes detected*	*Median UMIs per cell*	*Total number of reads*	*Percent mapped reads*	*Cells passed QC*	*Yield*
S1-P0	14,054	36,086	2,985	21,313	11,538	507,158,850	91.5%	13,667	97.25%
S1-P7	21,351	24,012	3,114	21,617	10,497	512,691,202	92.5%	20,454	95.80%
S2-P0	15,895	31,837	2,937	21,455	10,265	506,060,345	91%	15,749	99.08%
S2-P7	18,814	25,772	3,134	21,375	11,003	484,877,152	90.7%	14,526	77.21%
S3-P0	3,900	148,075	4,733	21,141	27,120	577,495,314	96.7%	3,191	81.82%
S3-P7	6,812	87,509	4,437	21,151	28,462	596,116,448	95.9%	5,509	80.87%
S4-P0	9, 222	63,442	3,325	21,896	15,917	585,063,284	96.9%	8,523	92.42%
S4-P7	5,767	99,463	4,737	20,969	33,249	573,605,784	95.6%	4,302	74.60%
S5-P0	9,207	62,505	3,253	22,071	15,662	575,484,333	96.1%	8,618	93.60%
S5-P7	7,501	78,123	4,264	21,291	26,063	586,007,207	95.6%	5,996	79.94%
S6-P0	9,696	61,136	3,386	22,090	15,712	592,778,459	96.2%	8,605	88.75%
S6-P7	7,414	76,300	4,306	20,969	25,506	565,693,408	94.8%	4,727	63.76%

Supplementary Table S2. Overview of the scRNA-seq data for samples.

Table S3.

Name	*Reference number*	*Supplier*	*Dilution*	*Application*
BAMBI	bs-12418R-Cy5	Bioss Antibodies	1:100-1:300	FACS, IF
BAMBI	bs-12418R	Bioss Antibodies	1:100-1:300	IF, WB
C3	sc-58926 AF488	Santa Cruz	1:200	IF
CCL2	66272-1-Ig	Proteintech	1:1,000	WB
CD3	563565	BD Biosciences	1:300	FCM, TC
CD3	16-0037-81	eBioscience	1:500	TC
CD4	553046	BioLegend	1:300	FCM
CD8	563068	BD Biosciences	1:300	FCM
CD8a	563068	BD Biosciences	1:300	FCM
CD21/CD35	123418	BioLegend	1:300	FCM
CD23	563988	BD Pharmingen	1:300	FCM
CD25	557192	BD Biosciences	1:300	FCM
CD28	16-0289-81	eBioscience	1:500	TC
CD38	102732	BioLegend	1:300	FCM
CD44	563971	BD Biosciences	1:300	FCM
CD45R/B220	103225	BioLegend	1:300	FCM
CD62 L	563252	BD Biosciences	1:300	FCM
CD69	104541	BioLegend	1:300	FCM
CD138	553714	BD Biosciences	1:300	FCM
Collagen II	ab34712	Abcam	1:200	IF
FoxP3	563101	BD Biosciences	1:300	FCM
GAPDH	60004-1-Ig	Proteintech	1:5,000	WB
GL7	144610	BioLegend	1:300	FCM
IgD	560868	BD Pharmingen	1:300	FCM
IgG	4408S	CST	1:1000	IF
IGFBP5	sc-515116	Santa Cruz	1:500	WB
Ki67	561283	BD Biosciences	1:100	FCM
MFGE8	ob388429	biorbyt	1:10-1:300	WB
Anti-mouse IgG Alexa Fluor 488	4408S	CST	1:1000	IF
Anti-mouse IgG Alexa Fluor 647	4410S	CST	1:1000	IF
Anti-rabbit IgG Alexa Fluor 488	4412S	CST	1:1000	IF

Supplementary Table S3. Antibodies used in this study. Abbreviation: FACS, Fluorescence-activated cell sorting; FCM, Flow cytometry; IF, Immunofluorescence; TC, Tissue culture; WB, Western blot.

Table S4.

Gene Name	*Forward Primer (5'-3')*	*Reverse Primer (5'-3')*
BAMBI	CGCCACTCCAGCTACATCTT	CAGTGGGCAGCATCACAGTA
CCL2	TGCAATCAATGCCCCAGTCA	GGGTCAGCACAGATCTCCTT
CEBPD	ACAGACCGTGGTGAGCTTG	GGCGATGTTGTTGCGCTC
COL1A1	CAAAGAAGGCGGCAAAGGTC	CACGCTGTCCAGCAATACCT
COL3A1	CCTTCGACTTCTCTCCAGCC	TTTCGTGCAACCATCCTCCA
DCN	GGCTGGACCGTTTCAACAGA	GATGGCATTGACAGCGGAAG
NEAT1	CACAGGCAGGGGAAATGTCT	TGCTGCGTATGCAAGTCTGA
FTH1	AGCTCTACGCCTCCTACGTT	CCTGAAGGAAGATTCGGCCA
GAPDH	TCAGTGGTGGACCTGACCTG	TGCTGTAGCCAAATTCGTTG
IGFBP3	GCCAGCGCTACAAAGTTGAC	ATGTGTACACCCCTGGGACT
IGFBP5	TCCCCACGTGTGTTCATCTG	AAATGGGATGGACTGAGGCG
MALAT1	TGGGGGAGTTTCGTACTGAG	TCTCCAGGACTTGGCAGTCT
MEST	TGGGAGCTCTTGCCTCTGTA	AGAATCGACACTGTGGACCG
MFGE8	TGTCTTCCCCTCGTACACCT	AGAAGGTCACACGCACAGAC
NUPR1	CCTTCCCACCAGCAACCAG	GGTAGGAATGGGCCAGGCTA
SERPINE2	GTCCTCGTCAACGCAGTGTA	GTCCTCGTCAACGCAGTGTA

Supplementary Table S4. Primers used in this study.

Supplementary legends for Data S1 to S2

Supplementary Data S1. Signature genes of C1-C3 clusters at P0.

Supplementary Data S2. Enrichment of the top 20 differentially expressed genes of C1 and C3 in P0 UC-MSC clusters with cell cycle regression.

Supplementary Data S3. Signature genes of C1-C3 clusters at P7.
